# Congenital chikungunya in a neonate with early‐onset sepsis and petechiae: An unusual case report

**DOI:** 10.1002/ccr3.6896

**Published:** 2023-01-23

**Authors:** Sakviseth Bin, Kimyi Phou, Sethikar Im

**Affiliations:** ^1^ Neonatal Intensive Care Unit Calmette Hospital Phnom Penh Cambodia

**Keywords:** chikungunya virus, congenital, mosquito‐borne disease, neonatal sepsis

## Abstract

Chikungunya, a mosquito‐borne disease, is posing threat to neonatal population in Cambodia because of its challenging diagnosis and insufficient attention compared with Dengue. In what follows, we present a case of serologically‐confirmed congenital chikungunya in a newborn presenting early‐onset sepsis, and the mother was underdiagnosed with intrapartum flu‐like infection.

## 1 INTRODUCTION

Chikungunya virus (CHIKV) is an arthropod‐borne virus that shares the same vectors, namely *Aedes albopictus and Aedes aegypti*, with dengue. In adults, CHIKV mostly causes two common clinical features including acute febrile arthralgia and maculopapular rash.^1^ Although vertical transmission is rare, during the outbreak in La Réunion, the chance of transmissibility, representing for 48.7%, was found high in the case of intrapartum viremia.^2^ Clinical manifestations in the neonatal period are reported to be variable and nonspecific.^3^ At the acute stage, the treatment is only supportive, and antivirals have not been proven to be effective.^4^

In Cambodia, CHIKV was first identified in 1961. In 2011, 24 sporadic cases, in which the patients' ages range from 2 to 56 years, were reported across the country.^5^ In 2012, there was an outbreak in a remote provincial village, and 190 of 425 residents (about 45%) were tested positive.^6^ However, neither neonatal infection nor mother‐to‐child transmission was reported.

In what follows, we report a rare case of serologically‐confirmed chikungunya infection in a 3‐day‐old neonate, born to a mother with intrapartum flu‐like syndrome. The girl was initially treated for early‐onset sepsis (EOS) and later suspected of mosquito‐borne congenital infection due to the new onset of eruptive fever and petechiae on Day 3 of life and progressive thrombocytopenia.

## 2 CASE PRESENTATION

### 2.1 Birth history

A female neonate was delivered at 38 + 6 weeks of gestation with a birth weight of 2250 g (below 3rd percentile) via vaginal delivery. The amniotic fluid was clear, and the Apgar scores were 8, 9, and 10 at 1, 5, and 10 min, respectively.

The 23‐year‐old primigest mother had no pregnancy‐related complications. Prenatal care was done regularly at a private clinic with unremarkable serology in the first semester: Hepatitis B, HIV, and syphilis negative. She had flu‐like syndrome and an episode of fever (38.5°C) 2 days prior to admission. At arrival, she was afebrile and COVID‐19 rapid antigen test was negative. She was admitted for unspecified sepsis during labor and treated with ampicillin for 5 days. During her stay, the initial investigation demonstrated an isolated elevated C‐reactive protein (CRP) of 130 mg/L. Complete blood count (CBC) was normal, with leucocytes 4.4 × 10^9^/L, platelets 223 × 10^9^/L, and hemoglobin 115 g/L. Urine examination was normal, and hemoculture later was proven negative. Dengue serology was negative. Few hours after admission, the delivery was spontaneous and uneventful.

### 2.2 Initial management

At Special Care Nursery, the infant was active and pink, with good sucking. The physical examination was unremarkable. Due to the possible risks of perinatal infection, a septic workup was done and showed a normal leucocyte count (11.4 × 10^9^/L), with an absolute neutrophil count of 7.2 × 10^9^/L, lymphocytes of 2.3 × 10^9^/L (20%) and immature to total neutrophil ratio (I/T ratio) 0.01; hemoglobin and platelets were within the normal range (Table 1). Inflammatory markers, however, were elevated: CRP of 34.64 mg/L and procalcitonin (PCT) of 1.48 mcg/L. Per unit protocol for suspected early‐onset sepsis, empiric antibiotics including Ampicillin and Cefotaxime were initiated.

**TABLE 1:** Laboratory investigations of the infant

	Day 0	Day 3	Day 5	Day 7	Day 10	Day 20
Leucocytes (4–9 × 10^9^/L)	11.4	16.4	29.28	28.5	13.8	19.3
Hemoglobin (130–170 g/L)	173	139	145	142	125	120
Platelets (150–450 × 10^9^/L)	169	78	60	66	112	185
C‐reactive protein (<5 mg/L)	34.64	43	18.77	10		2.3
Procalcitonin (<0.5 mcg/L)	1.48					
Culture						
Blood culture	Neg	Neg	Neg			
Gastric aspirate	Neg					
Cerebrospinal fluid		Neg				
Bilirubin total/direct (<215/<3.4 μmol/L)			21/9.4			
AST (<31 U/L)			120			60
ALT (<34 U/L)			19			15
BUN (4–13 mmol/L)			8.2			
Creatinine (44–106 μmol/L)			53			
Calcium (2–2.5 mmol/L)			2.4			
Sodium (135–148 mmol/L)			147			
Potassium (3.5–5.2 mmol/L)			4.1			
Chloride (97–107 mmol/L)			112			

**FIGURE 1:**
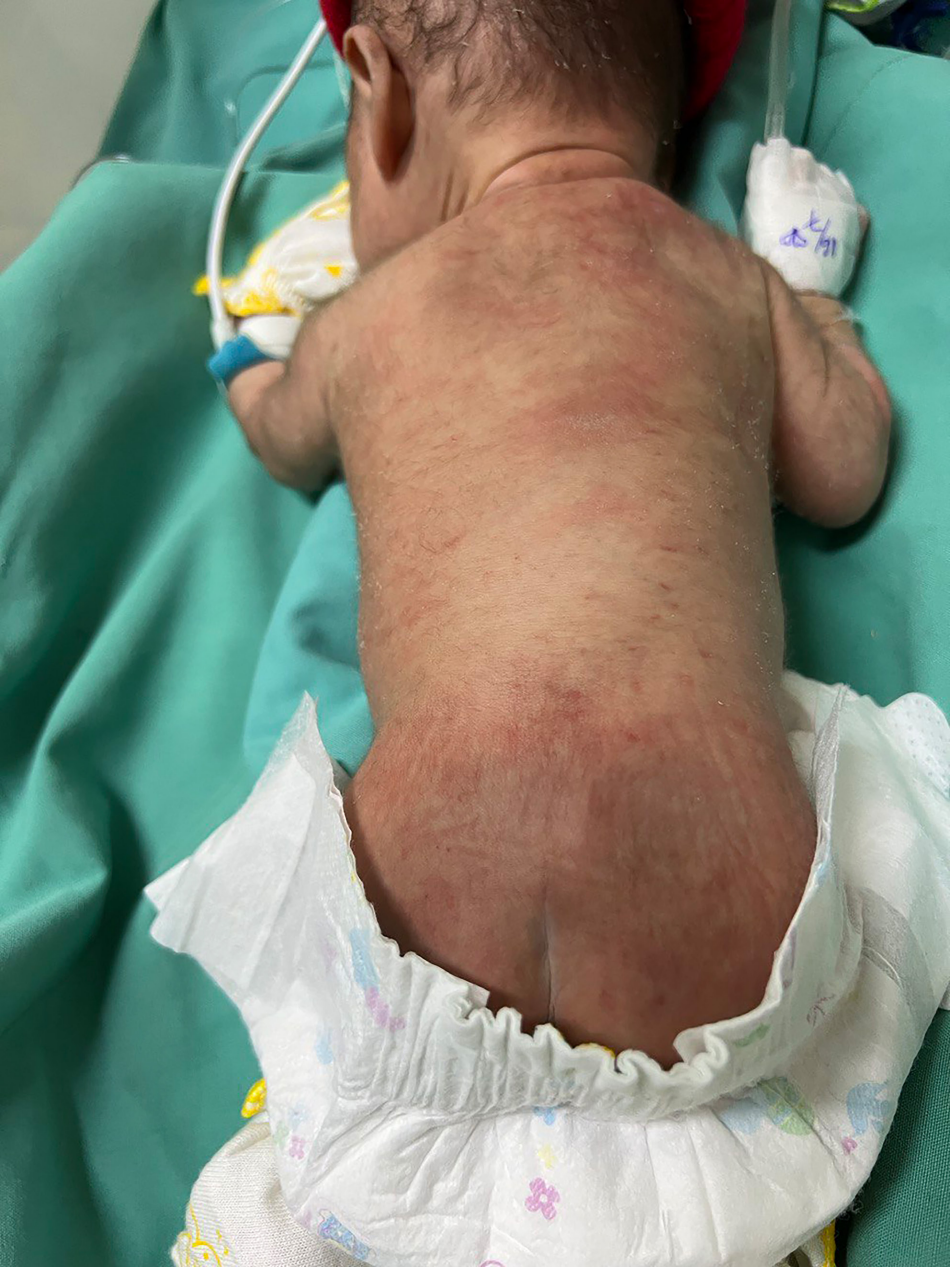
Petechiae on the back (Day 3)

At Day 3 of life, the infant started to develop morbilliform rash with petechiae, predominantly found over the back (Figure 1), and intermittent nocturnal fever (38°C). Vital signs were stable. There were neither neurological signs nor hepatosplenomegaly. No mosquito bite marks were reported. Control blood tests showed decreased platelets (78 × 10^9^/L) and increased CRP (43 mg/L). Syphilis tests were nonreactive. Hemoculture and gastric aspirate done at admission were negative. Brain ultrasound, done before Lumbar Puncture (LP), showed no bleeding signs. Heart ultrasound showed no defect. The second blood culture was drawn, and LP was done, with normal results: leucocytes 06/mm^3^, albumin 0.5 g/L, and glucose 0.79 g/L. Based upon local guidelines, the clinicians upgraded the antibiotics to second line: Meropenem and Amikacin.

About 48 h later, because of the persistently on–off fever and progressively decreasing platelets, we interviewed parents by phone during the restricted period amid COVID pandemic to get more detailed information before any further investigations. According to the mother about 2 days after delivery, she started developing rash, with low‐grade fever and persistent arthralgia. Given the information, we decided to rule out other mosquito‐borne congenital infections, in an early stage, by combined PCR, which later confirmed the diagnosis of congenital chikungunya (Table 2).

**TABLE 2:** Virology report

Combined detection of Dengue/Zika/Chikungunya		
Method	Multiplex real‐time RT‐PCR	
Results	Zika	Not detected
	Dengue	Not detected
	Chikungunya	Detected
Conclusion	Presence of Chikungunya viral RNA Infection with Chikungunya virus confirmed	

### 2.3 Differential diagnosis

1. Early‐onset neonatal sepsis, culture not proven
2. Hypoproliferative thrombocytopenia, associated with small‐for‐gestational‐age neonates
3. Mosquito‐borne congenital infection such as congenital dengue

### 2.4 Outcome and follow‐up

The treatment was supportive. Apyrexia was noted on day 6 of life. Antibiotics were discontinued 7 days after the negativation of the second blood culture. Platelets increased progressively to 112 × 10^9^/L at the discharge (Day 10). She was brought for a follow‐up on day 20, with good weight gain and normalized platelets. The girl will be follow up every 3 months until the age of 2 years, following our local follow‐up program for low birth weight.

## 3 DISCUSSION

Mother‐to‐child transmission of Chikungunya was first described in La Réunion during the outbreak between 2005 and 2006, during which 19 out of 39 neonates from the mothers with intrapartum viremia were infected, corresponding to the transmission rate of 48.7%. Maternal infection was considered intrapartum when the symptoms appeared 2 days prior to the delivery and 2 days after.^2^

Most infected newborns are asymptomatic at birth, and symptoms appear mostly after 3 days of life.^2^, ^7^ The prospective French study showed that the 19 cases presented with fever, poor feeding, and pain, while petechiae were reported in 47.3% and rubella‐like rash in 52.6% of the cases. The most common laboratory findings included thrombocytopenia (89.4%), of which 47.3% were severe, lymphopenia (68.4%), mild transaminitis (52.6%), and hypocalcemia (47.3%).^2^ Later study conducted in four different regions in Latin America during 2014–2015 showed similar clinical features, in which fever, poor feeding, and irritability were the commonest in 169 neonates with CHIKV.^7^

Although Chikungunya is a self‐limited viral disease in healthy adults, young children and the elderly with co‐existing conditions are considered high‐risk population.^1^ For example, 52.6% of the infected neonates in La Réunion were severe. There were two complications observed: Encephalopathy with cerebral edema confirmed by MRI was diagnosed in nine cases (90%) and hemorrhagic fever in only one case (10%). Eight cases (80%) required mechanical ventilation with a mean duration of 7 days, and about two‐thirds were in shock status. All children, however, survived; persistent disabilities were reported in four cases (45%) of newborns with neurological complications.^2^

Chikungunya virus serology, specifically IgM, can be used for the diagnosis; however, it is challenging in neonates since the virus is detected from about 5 days (range 1–12 days) following the onset of symptoms.^1^ RT‐PCR is the confirmatory test recommended by the Centers of Disease Control and Prevention (CDC) to diagnose the infection in an early stage, during the first 8 days following the onset of the disease,^8^ with very high sensitivity and specificity of 100% and 98.4%, respectively.^9^

The treatment in the neonatal period relies on supportive care, consisting of close monitoring (body temperature, feeding, pain, skin condition, and hydration), antipyretics, and fluid management. Antivirals have no indication.^4^

In our case, the neonate was asymptomatic at birth with normal CBC and increased acute phase reactants. She developed intermittent fever, rash, and petechiae, with moderate thrombocytopenia on day 3 of life. Vertical mosquito‐borne infection was not initially considered due to the negativity of mother's dengue serology and the local burden of bacterial early‐onset sepsis. Syphilis tests were done due to the resurgence of congenital syphilis in our setting.^10^, ^11^ Congenital chikungunya was later diagnosed with RT‐PCR (for Dengue, Chikungunya and Zika). The infant was discharged home on day 10 of life with normal neurological examination.

There are few limitations to our case report. First, there are no MRI in our facility to fully evaluate the neurological involvement. However, brain ultrasound showed no lesions, and normal LP ruled out neurological involvement. Second, antibiotics were used even after the confirmatory diagnosis and stopped only after 7 days of the negativation of the second hemoculture. It was for safety coverage due to severe sepsis. Lastly, out of financial concern, only PCR was done in the neonate on Day 5. Serology might be nonreactive in such an early stage, and together, it costs a lot.

## 4 CONCLUSION

Chikungunya neonatal infection is rare, which makes the diagnosis quite challenging. A careful maternal history plays a leading role in suspecting the possible perinatal infection. In a dengue‐endemic country, congenital arboviral infections, basically CHIKV, should be suspected and ruled out in case of early‐onset sepsis not corresponding to conventional antibiotics in neonates born to the mothers from dengue‐endemic regions with flu‐like syndrome around delivery time.

## AUTHOR CONTRIBUTIONS

**Sakviseth Bin:** Conceptualization; investigation; methodology; validation; writing – original draft; writing – review and editing. **Kimyi Phou:** Investigation; writing – review and editing. **Sethikar Im:** Supervision; writing – review and editing.

## FUNDING INFORMATION

There was no funding support for the case report.

## CONFLICT OF INTEREST

None declared.

## ETHICAL APPROVAL

No ethical approval is required.

## CONSENT

Written informed consent was obtained from the patient's parents and is available for review upon request.

## Data Availability

The data that support the findings of this study are available from the corresponding author upon reasonable request.
